# The relationship between biochemical recurrence and number of lymph nodes removed during surgery for localized prostate cancer

**DOI:** 10.1186/s12894-023-01228-3

**Published:** 2023-04-28

**Authors:** Paul Doan, Athos Katelaris, Matthijs J. Scheltema, Andrew Hayen, Amer Amin, Amila Siriwardana, Minh Tran, Bart Geboers, William Gondoputro, Anne Maree Haynes, Jayne Matthews, Warick Delprado, Phillip D. Stricker, James Thompson

**Affiliations:** 1 St. Vincent’s Prostate Cancer Research Centre, Department of Urology, Darlinghurst, Sydney, NSW Australia; 2 Garvan Institute of Medical Research & The Kinghorn Cancer Centre, Darlinghurst, 384 Victoria St, 2010 NSW Australia; 3 Departments of Urology and Radiology and Nuclear Medicine, Amsterdam University Medical Centers (location VUmc), Amsterdam, the Netherlands; 4 Australian Centre for Public and Population Health Research, University of Technology, Sydney, NSW Australia; 5 Douglas Hanly Moir Pathology, Sydney, NSW Australia

**Keywords:** Prostate cancer, Lymph node dissection, Biochemical recurrence

## Abstract

**Purpose:**

To assess whether completeness of pelvic lymph node dissection (PLND) as measured by lymph node yield reduces biochemical recurrence (BCR) in men undergoing radical prostatectomy (RP) for prostate cancer (PCa), stratified according to Briganti nomogram-derived risk (≥5% vs. < 5%) of lymph node invasion (LNI).

**Methods:**

Retrospective study of 3724 men who underwent RP between January 1995 and January 2015 from our prospectively collected institutional database. All men included had minimum five years follow-up and were not given androgen deprivation therapy or radiotherapy prior to BCR. Primary endpoint was time to BCR as defined by PSA > 0.2ng/ml. Patients were analysed according to Briganti Nomogram derived risk of ‘low-risk’ (< 5%) vs. ‘high-risk’ (≥ 5%). Extent of PLND was analysed using number of nodes yielded at dissection as a continuous variable as well as a categorical variable: Group 1 (limited, 1–4 nodes), Group 2 (intermediate, 5–8 nodes) and Group 3(extensive, ≥9 nodes).

**Results:**

Median follow-up in the overall cohort was 79.7 months and 65% of the total cohort underwent PLND. There were 2402 patients with Briganti risk of LNI < 5% and 1322 with a Briganti risk of LNI ≥5%. At multivariate analysis, only PSA (HR1.01, p < 0.001), extracapsular extension at RP (HR 1.86, p < 0.001), positive surgical margin (HR 1.61, p < 0.001) and positive lymph node on pathology (HR 1.52, p = 0.02) were independently associated with BCR. In the high-risk group, increased nodal yield at PLND was associated with reduction in risk of BCR (HR 0.97, 95%CI 0.95-1.00 p = 0.05, Cochran Mantel Haenszel test, p < 0.05: respectively). In the low-risk group increased number of nodes at PLND did not reduce risk of BCR.

**Conclusions:**

In this study of extent of PLND at RP, higher nodal yield did not reduce risk of BCR in low-risk men (Briganti risk < 5%), however there was a weak benefit in terms of reduced long-term risk of BCR in high-risk men (Briganti risk ≥5%).

**Supplementary Information:**

The online version contains supplementary material available at 10.1186/s12894-023-01228-3.

## Introduction

There is conflicting evidence surrounding the therapeutic value of pelvic lymph node dissection (PLND) at radical prostatectomy (RP) for the treatment of localized prostate cancer (PCa) [1–4]. Current EAU guidelines recommend that PLND is performed in higher-risk PCa patients when the estimated risk for lymph node involvement is greater than 5% on preoperative nomograms [5–7]. PLND is not recommended in patients with a low risk of lymph node involvement due to the inherent morbidity [8]. A recent metanalysis has shown that the extent of PLND was associated with increased intraoperative and postoperative complications, primarily lymphoceles [9]. On the contrary, it has been suggested that patients with a high risk of lymph node invasion (LNI) could potentially benefit from PLND by reducing micro-metastatic disease [10]. Greater extent of lymph node dissection was associated with lower risk of prostate cancer-specific death at 10 years in selected studies [11]. However, other large retrospective studies [12], RCTs [13, 14] and meta-analyses have all found no oncologic benefit for extended PLND over limited PLND or no PLND [15].

Of the two RCTs recently published regarding ePLND vs. limited PLND, Lestingi et al. included a very high proportion of patients with low to intermediate (ISUPGG 1–2 disease 74–79% of patients) which were very unlikely to benefit from PLND regardless of extent. Further, in the RCT by Touijer et al. there was minimal difference in median lymph node yield between limited PLND (12 nodes) and ePLND (14 nodes) indicating no significant difference in PLND template intraoperatively. We believe that to correctly assess the therapeutic benefit of PLND, the extent of template as well as the completeness of lymph node clearance, demonstrated by higher lymph node yields, are both vitally important.

RCTs are underway to address this important clinical question, but these studies will take more than a decade to complete enrolment and follow-up, thus we must rely on data from the highest quality retrospective studies in the interim.

The aim of this study was to assess whether the completeness of PLND as measured by lymph node yield reduces biochemical recurrence (BCR) in men undergoing RP for PCa, stratified according to Briganti nomogram-derived risk (≥5% vs. < 5%) of lymph node invasion.

## Patients and methods

### Study population

This retrospective observational cohort study was approved by our Institutional Review Board (HREC12/231). We identified 4181 men who underwent RP between January 1995 and January 2015 from our institutional database. Inclusion criteria were: (a) all men who underwent RP with a minimum of 5 years follow up, (b) no standardized adjuvant androgen deprivation therapy (ADT) or radiotherapy after RP but before BCR, (c) initial biochemical response to RP of PSA < 0.2 and (d) availability of complete follow up data. Patients were excluded if their follow-up was incomplete, BCR status was unclear/unknown, PLND status was unknown, or if they had received adjuvant ADT or radiotherapy prior to BCR. A total of 3724 patients were included for analysis.

### Surgical approach

Radical prostatectomy was performed using an open retropubic, or robotic assisted laparoscopic, approach. The standard PLND template at our institution included removal of external iliac, internal iliac and obturator LNs. Lymph node yield varied dependent on difficulty of case, surgeon preference and risk of LNI. Patients were selected for PLND mostly through utilizing the MSKCC and Briganti nomograms [5, 6].

### Clinical characteristics

All patients clinical and pathological data was collected. This included age, PSA at diagnosis, clinical stage, pathological tumour stage, surgical margin status, number of lymph nodes examined and number of lymph nodes positive for metastases. Radical prostatectomy specimens and lymph node specimens were potted separately and reviewed by specialists uro-pathologists.

Standard follow up protocols involved 3-monthly PSA measurements, and in the case of BCR staging imaging was performed using a bone scan/CT or Choline PET scan or PSMA PET scan.

### End point

The primary endpoint was biochemical recurrence defined as a PSA > 0.2 ng/mL. Time to BCR was calculated as the time from RP to BCR or last follow-up.

### Statistical analysis

For statistical analysis all patients were stratified into risk of LNI based on the Briganti Nomogram: low risk (< 5% risk) vs. high risk (≥ 5% risk) [5]. Patients were then grouped into lymph node yield at dissection: Group 1 (limited, 1–4 nodes), Group 2 (intermediate, 5–8 nodes) and Group 3(extensive, ≥9 nodes).

Univariate + multivariate Cox proportional hazard regression models were used to test the relationship between BCR and number of nodes removed, positive nodes, margin status, extracapsular extension, and other clinical factors. The variable of interest (lymph node yield) was modelled as both a continuous and categorical variable (1–4, 5–8, > 9) for the development of BCR. Kaplan-Meier analysis using stratified KM curves and the log-rank test to compare groups were performed to graphically depict the BCR-free, cancer-specific, and overall survival per node or Briganti risk group. Statistical analysis was performed using SAS v9.4, p < 0.05 was considered significant.

## Results

### 1. Baseline characteristics and overall oncologic outcomes

Baseline clinico-pathologic characteristics of the study population are shown in Table 1. 3724 men who underwent RP between 1995 and 2015 were included in the analysis. Median follow up in the overall cohort was 79.7 months (IQR 50.8-117.3mo).

The overall cohort was divided by Briganti risk of lymph node invasion, with 2402 having a Briganti risk of LNI < 5% and 1322 having a Briganti risk of LNI ≥5%.

Overall, 698 (18.7%) men experienced BCR, comprising 414 (31%) Briganti high-risk patients and 284 (11.8%) Briganti low-risk patients at a median of 29.7 (IQR 12.9–54.4) and 35 (IQR 16.9–70.1) months post-RP respectively. 158 (12%) Briganti high-risk and 72 (3%) Briganti low-risk patients received adjuvant treatment.

**Table 1:** Baseline Patient Clinical and Pathological Characteristics

	Briganti Low Risk (n = 2402)	Briganti High Risk (n = 1322)	Overall cohort (N = 3724)
Age at RP (SD)	60.7 (6.7)	62.3 (6.6)	61.3 (6.7)
Biopsy Gleason score (%)			
≤ 6 7 8 9–10	1101 (45.8) 1248 (52.0) 34 (1.4) 19 (0.8)	132 (10) 881 (66.6) 146 (11.0) 163 (12.3)	1233 (33.1) 2129 (57.2) 180 (4.8) 182 (4.9)
Preoperative PSA ng/mL (SD)	6.8 ( 3.7)	9.8 ( 9.4)	7.9 (6.5)
Clinical T stage			
cT1 (%) T1a T1b T1c cT2 (%) T2a T2b T2c cT3 (%) T3a T3b	1530 (63.7) 38 22 1470 872 (36.3) 567 165 140 0	347 (26.2) 6 3 338 891 (67.4) 437 288 166 84 (6.4) 74 10	1877 (50.4% 44 25 1808 1763 (47.3) 1004 453 306 84 (2.3) 74 10
Surgical margins, n (%)			
Negative Positive	1846 (76.9) 556 (23.1)	877 (66.3) 445 (33.7)	2723 1001
ECE status			
Negative Positive	1664 (69.3) 738 (30.7)	511 (38.7) 811 (61.3)	2175 1549
Pathological Gleason score (RP)			
≤ 6 7 8 9–10	635 (26.4) 1674 (69.7) 42 (1.8) 51 (2.1)	95 (7.2) 897 (67.9) 99 (7.5) 231 (17.4)	730 2571 141 282
Pathological T stage, n (%)			
pT2 pT3	2319 (96.6) 83 (3.4)	1090 (82.5) 232 (17.5)	3409 (91.5) 315 (8.5)
Lymph node dissection status n (%)			
Positive Negative No lymph node dissection performed	7(0.3) 987 (41.1) 1408 (58.6)	67 (5.1) 978 (74.0) 277 (20.9)	74 (2.0) 1965 (52.8) 1685 (45.2)
D’Amico risk group n (%)			
Low Intermediate High	722 (30.1) 1314 (54.7) 366 (15.2)	0 320 (24.2) 1002 (75.8)	722(19.4) 1634 (43.9) 1368 (36.7)
Lymph nodes examined Median (SD)	1.6 (2.8)	4.1 (4.9)	2.5 (3.9)
Lymph nodes removed n (%)			
1-4 5-9 10+	746 (75) 161 (16.2) 87 (8.8)	653 (62.5) 219 (21) 173 (16.5)	1399 (68) 380 (18.6) 260 (12.8)
Patients receiving any adjuvant treatment, n (%)			
Yes	72 (3%)	158 (12%)	230 (6.2)
Patients receiving Neoadjuvant HT, n (%)			
Yes	23 (1%)	106 (8%)	129

### 2. Predictors of biochemical recurrence in the overall cohort

Univariate cox regression analysis demonstrated multiple factors to be predictors of biochemical recurrence, including the number of nodes excised (HR 1.06, p < 0.001)(Supplementary Table 1). At multivariate analysis, only PSA (HR1.01, p < 0.001), ECE at RP (HR 1.86, p < 0.001), positive surgical margin (HR 1.61, p < 0.001) and positive lymph node on pathology (HR 1.52, p = 0.02) were independently associated with biochemical recurrence. Number of nodes excised was *not* associated with BCR in the overall cohort including both low and high Briganti risk patients.

### 3. Extent of pelvic lymph node dissection on BCR in those with high risk of lymph node invasion

1322 men had a Briganti nomogram risk of LNI ≥5%. 1045 (79%) of these men underwent PLND. Using lymph node yield as a continuous variable, multivariate analysis demonstrated that an increased number of lymph nodes removed at PLND was associated with a borderline reduction in the risk of BCR (HR 0.97, 95%CI 0.95-1.00 p = 0.05) (Table 2.).

**Table 2:** Multivariate analysis of lymph node yield as a continuous variable for men with high risk of LNI ≥5%

n = 1045	HR	95% CI	P value
Number of nodes taken	0.97	0.95-1.0	0.05
PSA	1.01	1.00-1.02	0.002
RP ECE	1.77	1.35–2.34	< 0.001
Margin status	1.27	1.03–1.57	0.03
RP ISUP score			
1 (ref)			
2 3 4 5	0.43 0.75 1.54 1.40	0.28–0.65 0.49–1.16 0.96–2.46 0.90–2.17	< 0.001 0.20 0.07 0.14
LN Positive	1.42	0.98–2.05	0.07

Similarly, when lymph node groups were categorically grouped, men who underwent larger lymph node dissections had significantly lower rates of BCR as shown in Table 3, with a significant trend towards lower risk of BCR with increased nodal yield (Cochran Mantel Haenszel test, p < 0.05).

**Table 3:** Multivariate analysis of lymph node yield as categorical variable for men with high risk of LNI ≥5%

n = 1045	HR	95% CI	P value
Nodes removed			
1–4 (ref)			
5–8 9+	0.74 0.72	0.56–0.97 0.51-1.00	0.03 0.05
PSA	1.01	1.00-1.02	0.001
RP ECE	1.77	1.35–2.34	< 0.001
Margin status	1.29	1.04–1.60	0.02
RP ISUP score			
1 (ref)			
2 3 4 5	0.43 0.75 1.54 1.40	0.28–0.65 0.49–1.16 0.96–2.46 0.90–2.17	< 0.001 0.20 0.07 0.14
LN Positive	1.42	0.98–2.05	0.07

**Fig. 1:**
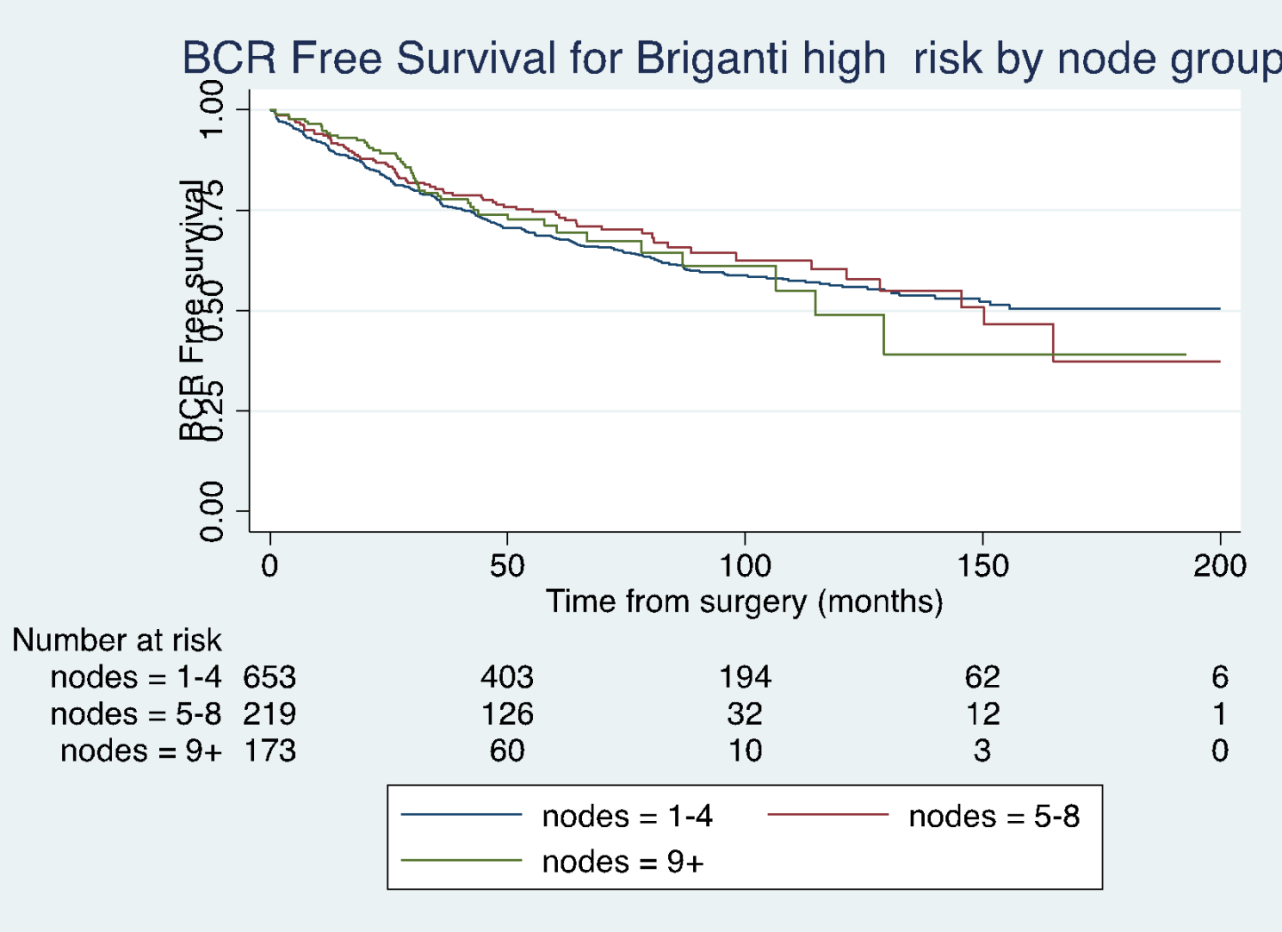
Kaplan-Meier analysis depicting BCR free survival for high-risk patients in different lymph node yield groups

**Fig. 2:**
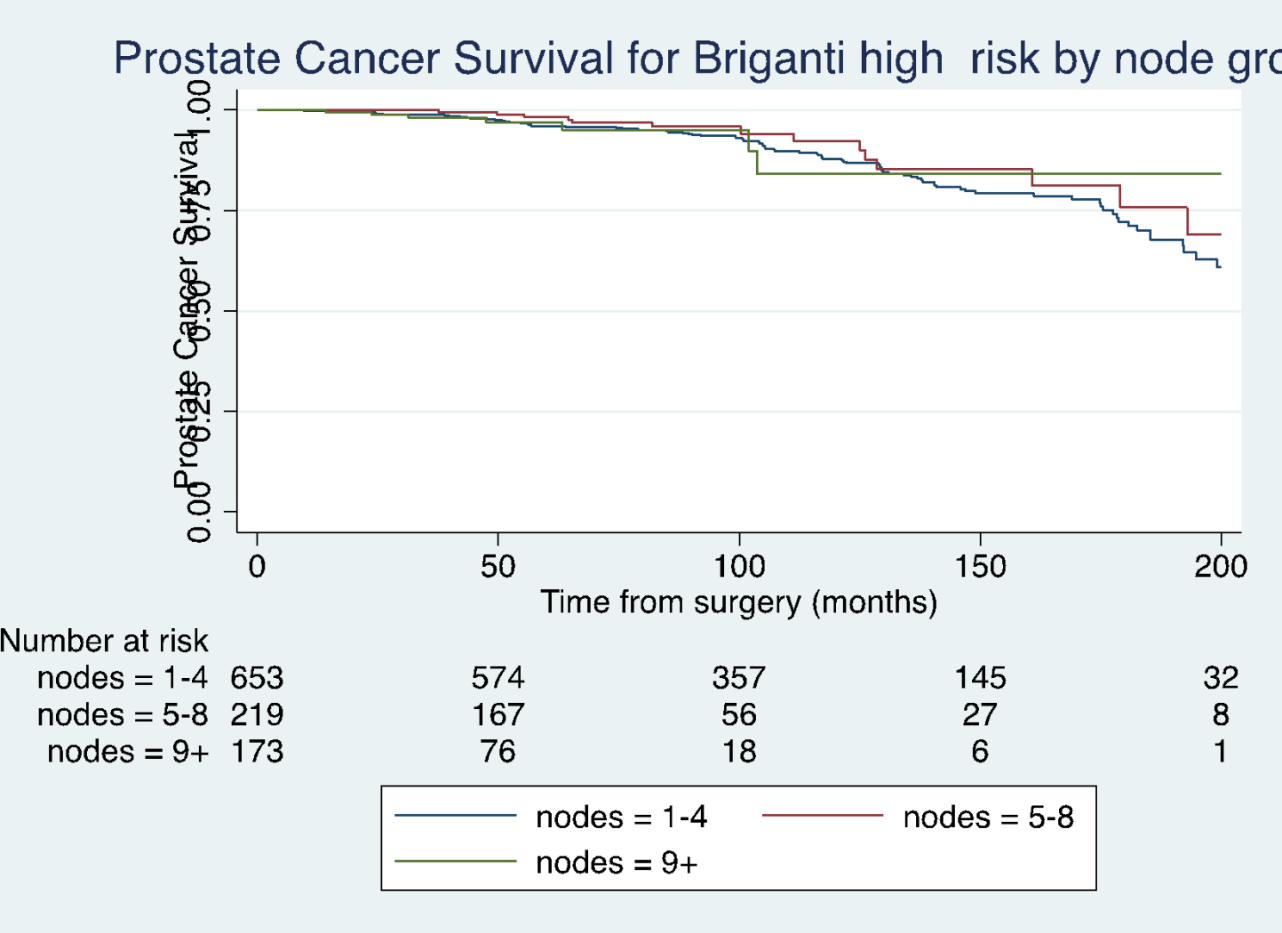
Kaplan-Meier analysis depicting cancer free survival for high-risk patients in different lymph node yield groups

**Fig. 3:**
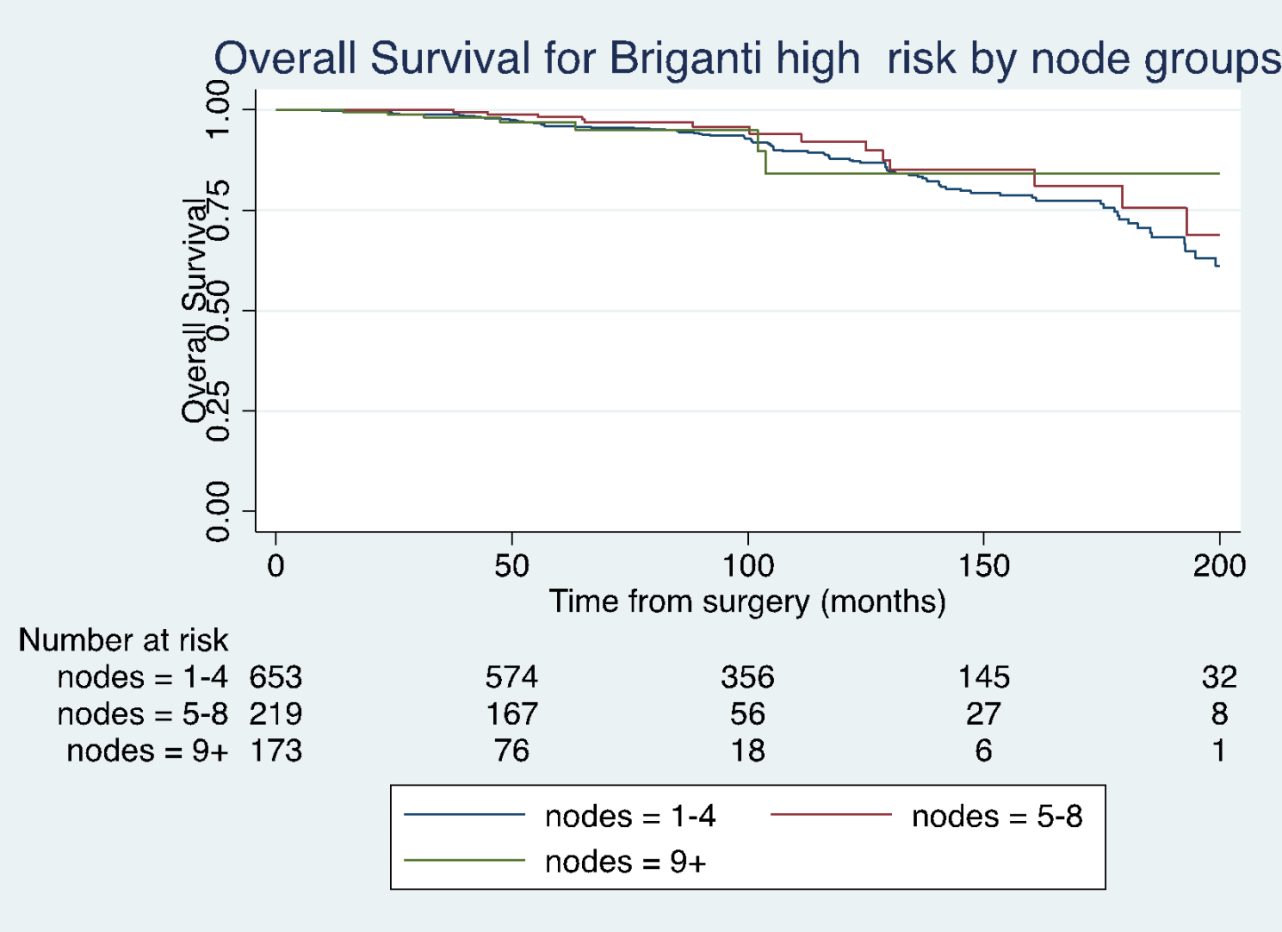
Kaplan-Meier analysis depicting overall survival for high-risk patients in different lymph node yield groups

Kaplan-Meier curves comparing the effect of lymph node yield on BCR-free survival, cancer specific survival and overall survival are shown in Fig. 1, 2 and 3. These results were not statistically significant (Log rank test, p > 0.05).

### 4. Extent of PLND on BCR in those with low risk of lymph node invasion

A total of 2402 men had a Briganti nomogram risk of LNI < 5%. 994 (41%) of these men underwent PLND. Using lymph node yield as a continuous variable, multivariate analysis demonstrated that increased number of lymph nodes removed at PLND was not significantly associated with a reduced risk of BCR (HR 0.97, 95%CI 0.92–1.03 p = 0.31). When the entire cohort of 2402 was analyzed (including those with nodes removed = 0) this effect again did not reach statistical significance (HR 1.03, 95% CI 0.99–1.07, p = 0.16) (Supplementary Table 2). Similarly, when lymph node groups were categorically grouped, men who underwent larger lymph node dissections did not have significantly lower rates of BCR in both groups on multivariable analysis (Supplementary Table 3).

## Discussion

This study analysed the relationship between lymph node yield and risk of cancer recurrence in men undergoing RP, stratified by pre-operative likelihood of nodal involvement. We found no difference in risk of BCR with increased nodal yield at RP in low-risk men (Briganti risk < 5%), however in high-risk men (Briganti risk ≥5%) there was a modest benefit in terms of reduced BCR risk at a median of 8-years follow-up, on multivariate analysis.

The present study adds incremental value to the existing evidence base, due to several strengths of the study which overcome many of the limitations of previous studies including a large sample size, long term follow-up (median 80 months), groups stratified to high-risk and low-risk according to the gold standard for prediction of LNI (Briganti nomogram risk), analysis of lymph node yield as a continuous and categorical variable as opposed to a theoretical standard PLND and ePLND template with multivariate analysis to adjust for confounding factors. The analysis of lymph node yield may represent a better surrogate for extent of PLND then theoretical templates and thus improve evaluation of the therapeutic benefit of PLND. Further, the data was collected from a prospectively maintained database with detailed baseline and follow-up data as compared to analysis performed using population wide registries such as the SEER or BAUS database.

Previous studies and meta-analyses have reported conflicting results regarding the oncologic benefit of performing PLND, but the weight of evidence is consistent with the findings from our study, demonstrating a consistent lack of benefit in low-risk men and conflicting evidence regarding a weak benefit for ePLND in high-risk men [10, 12, 13, 16–20]. For example, in the recent RCT by Lestingi et al., there was no benefit to extended vs. limited PLND in the overall population, but in the subgroup with ISUP grade 3–5 disease, extended PLND had a significantly better BCR free survival (HR 0.33, 95% CI 0.14–0.74, interaction p = 0.007). This finding was hypothesis-generating rather than definitive (due to small sub-group sample size) but is nevertheless consistent with the results of the present study, wherein only the high-risk sub-group appear to benefit from increased lymph node yield.

In the current multivariate analysis, clinical factors including PSA, ECE, surgical margin and positive nodes were independently associated with BCR. Increased lymph node yield was associated with reduced risk of BCR on multivariate analysis only in the high-risk subgroup, but not in the overall cohort or the low-risk subgroup. A retrospective multi-variate analysis of 9,742 patients from 4 institutions with Briganti risk > 5% by Preisser et al. showed that PSA, surgical margins, grade and pathological stage were independent predictors of BCR, however ePLND with a median yield of 14 nodes (vs. no PLND) was not a predictor of BCR, metastasis or PCa-death [12]. However, this study was limited by a short median follow-up of 33.5 months and did not assess whether higher vs. lower lymph node yield influenced BCR. In a systematic review of 66 studies, Fossati et al. demonstrated no significant benefit for PLND against no PLND in terms of BCR, metastasis free survival or cancer specific survival. However, many of the studies were limited by lack of multivariate analysis, failure to stratify into lower- vs. higher risk groups, short median follow-up, and small sample size.

Our finding that increased nodal yield reduced BCR risk, is supported by other studies. A meta-analysis of 6 studies involving 5,554 patients who underwent PLND demonstrated that increased lymph node yield with ePLND had a significant benefit in terms of BCR-free survival (HR 0.62, 95%CI 0.36–0.87)[21]. Choo et al., in a meta-analysis of 9 studies reported that there was a significant difference in BCR for ePLND vs. limited PLND, thus demonstrating the potential oncological benefit of increased lymph node yield (HR 0.71, 95% CI 0.56–0.90; p = 0.005) [18].

If ePLND does ‘truly’ confer a very weak oncologic benefit in high-risk Pca, as in the present study (i.e. a slight reduction in BCR-risk at 8-years, with no difference in mestastasis or mortality), then this marginal benefit must be weighed against the cost and morbidity of ePLND. A recent metanalysis of the peri-operative outcomes found that overall, 14.1% of patients experienced at least one postoperative complication secondary to PLND [9]. Further, the pooled meta-analysis demonstrated that RP + standard PLND had significantly decreased risk of intra-operative and post-operative complications when compared to ePLND. Therefore, ePLND also confers a significant cost and resource burden on the healthcare system, given it adds up to 60–75 min to overall procedure duration and causes increased complications. Thus, there is an urgent need for an adequately powered, non-inferiority RCT of ePLND vs. no PLND in men undergoing RP for high risk Pca, combining a long-term PCa-survival primary endpoint with secondary oncologic, morbidity, QOL and cost endpoints, and ideally incorporating pre-operative PSMA-PET/CT (to guide ePLND beyond the template if positive, but not to guide selection for ePLND).

This study has limitations. The main limitation is its retrospective and non-randomised design. Whilst RCTs provide the ideal level of evidence to guide practice, those published to date have failed to resolve the therapeutic dilemma regarding the benefit of ePLND in high-risk men. Further trials of ePLND vs. no PLND in high-risk PCa with long-term follow-up are being planned or underway,[22] but will not report final outcomes for more than a decade.

A further limitation of our study is that only 12.8% of men who theoretically underwent an ePLND had ≥ 10 nodes removed as is seen in Table 3, which is fewer than expected. This may at least partially explain our modest (although still statistically significant) reduction in BCr in the high-risk group.

Furthermore, ^68^Ga-prostate-specific membrane antigen (PSMA) PET/CT has been shown to improve accuracy over conventional CT for staging of pelvic nodal disease in patients with high-risk prostate cancer [23], although PSMA-PET has limited sensitivity ≤ 50% for micrometastases < 4 mm [24, 25]. Given it is this sub-group of men with 1–2 micro-metastases who may in fact be most likely to achieve prolonged BCR-free survival from ePLND, using PSMA-PET-CT to guide selection for ePLND may result in avoidance of PLND in those men who are most likely to benefit from PLND. Conversely, men with positive PSMA-PET/CT for nodal disease are more likely to harbour distant micro metastatic disease and therefore receive reduced oncological benefit from PLND despite its morbidity. Thus, PSMA-PET should not be used to guide selection for PLND outside of RCTs. The benefit of PSMA-PET may be via combination with novel intra-operative PLND techniques such as PSMA-Tc radio-guided targeted PLND, PSMA-PET may augment the sensitivity of PLND via localising nodes outside a surgeon’s standard template (e.g. pre-sacral, meso-rectal, etc.), thus optimising the oncologic benefit of PLND, as seen in preliminary results from the DETECT trial at our institution [26, 27].

## Conclusions

In this study of PLND at RP, higher nodal yield did not reduce risk of BCR in low-risk men (Briganti risk < 5%), however there was a weak benefit in terms of reduced long-term risk of BCR in high-risk men (Briganti risk ≥5%). Thus, more extensive, thorough PLND may benefit men with high-risk prostate cancer.

## Electronic supplementary material

Below is the link to the electronic supplementary material.

Supplementary Material 1

## Data Availability

The datasets used and/or analysed during the current study available from the corresponding author on reasonable request.
