# Supplementary material for: The relationship between biochemical recurrence and number of lymph nodes removed during surgery for localized prostate cancer
Source: BMC Urol. 2023 Apr 28;23:68. doi: 10.1186/s12894-023-01228-3 (PMC10148506; doi:10.1186/s12894-023-01228-3)
Supplement: Supplementary file 1 — Supplementary Material 1 [file 12894_2023_1228_MOESM1_ESM.docx]

Supplementary Materials

**Supplementary Figure 1. Univariate Cox Proportional Hazard Regression model plotting BCR-Free Survival by Briganti Group**

**Supplementary Figure 2. Univariate Cox Proportional Hazard Regression model plotting BCR-Free Survival by Node Group**

**Supplementary Figure 3. Univariate Cox Proportional Hazard Regression model plotting BCR-Free Survival for Briganti Low Risk Group**

**Supplementary Figure 4. Univariate Cox Proportional Hazard Regression model plotting BCR-Free Survival for Briganti High Risk Group**

**Supplementary Table 1. Univariate and multivariate cox regression of clinical characteristics predicting biochemical recurrence**

| N=3724 | Univariate analysis | | | Multivariate analysis | | |
| --- | --- | --- | --- | --- | --- | --- |
|  | HR | 95% CI | P value | HR | 95% CI | P value |
| Number of nodes taken | 1.06 | 1.05-1.08 | <0.001 | 1.01 | 0.99-1.03 | 0.34 |
| Age | 1.03 | 1.01-1.04 | <0.001 | NS | NS | NS |
| PSA | 1.02 | 1.02-1.02 | <0.001 | 1.01 | 1.01-1.02 | <0.001 |
| Clinical stage  T1 (ref)  T2  T3 | 1.76  5.25 | 1.50-2.06  3.78-7.28 | <0.001  <0.001 | NS  NS | NS  NS | NS  NS |
| Pathological stage  1 (ref)  2  3 | 2.57  6.80 | 2.17-3.05  5.56-8.31 | <0.001  <0.001 | NS  NS | NS  NS | NS  NS |
| ECE | 3.32 | 2.83-3.89 | <0.001 | 1.86 | 1.54-2.24 | <0.001 |
| Positive margin | 2.27 | 1.95-2.63 | <0.001 | 1.61 | 1.37-1.88 | <0.001 |
| Positive Lymph Node | 4.97 | 3.60-6.84 | <0.001 | 1.52 | 1.07-2.15 | 0.02 |
| Neoadjuvant Hormones | 2.71 | 2.03-3.61 | <0.001 | NS | NS | NS |
| Any adjuvant treatment | 3.26 | 2.66-3.99 | <0.001 | NS | NS | NS |
| ISUP score at Biopsy  1 (ref)  2  3  4  5 | 1.26  3.06  5.11  4.67 | 1.02-1.56  2.47-3.79  3.93-6.66  3.49-6.23 | 0.027  <0.001  <0.001  <0.001 |  |  |  |
| ISUP score at RP  1 (ref)  2  3  4  5 | 1.19  2.73  5.91  6.77 | 0.93-1.52  2.12-3.52  4.33-8.06  5.19-8.83 | 0.15  <0.001  <0.001  <0.001 |  |  |  |

**Supplementary table 2. Multivariate analysis of lymph node yield as a categorical variable for men with LNI risk <5%**

| n=2402 (cat) | HR | 95% CI | P value |
| --- | --- | --- | --- |
| Nodes removed  0 (ref)  1-4  5-8  9+ | 1.67  1.52  1.18 | 1.27-2.20  0.99-2.34  0.59-2.36 | <0.001  0.06  0.65 |
| PSA | 1.06 | 1.04-1.10 | <0.001 |
| RP ECE | 1.54 | 1.18-2.02 | 0.002 |
| Margin status | 2.28 | 1.77-2.95 | <0.001 |
| RP ISUP score  1 (ref)  2  3  4  5 | 1.20  1.89  2.65  3.22 | 0.88-1.64  1.30-2.75  1.38-5.11  1.80-5.76 | <0.001  <0.002  <0.001  <0.001 |
| LN Positive | 6.00 | 2.17-16.64 | 0.001 |

**Supplementary Table 3. Multivariate analysis of lymph node yield as a categorical variable for men with LNI risk <5% (excluding those with zero nodes removed)**

| n=994 (cat) | HR | 95% CI | P value |
| --- | --- | --- | --- |
| Nodes removed  1-4 (ref)  5-8  9+ | 0.89  0.70 | 0.59-1.33  0.36-1.39 | 0.57  0.31 |
| PSA | 1.06 | 1.03-1.10 | <0.001 |
| RP ECE | 1.59 | 1.15-2.20 | <0.005 |
| Margin status | 1.93 | 1.41-2.62 | <0.001 |
| RP ISUP score  1 (ref)  2  3  4  5 | 0.91  1.33  1.95  1.97 | 0.63-1.31  0.86-2.05  0.95-4.03  0.96-4.07 | 0.60  0.20  0.07  0.07 |
| LN Positive | 5.59 | 2.00-15.62 | 0.001 |
